# Structure, organization and evolution of ADP-ribosylation factors in rice and foxtail millet, and their expression in rice

**DOI:** 10.1038/srep24008

**Published:** 2016-04-21

**Authors:** Mehanathan Muthamilarasan, Venkata R. Mangu, Hana Zandkarimi, Manoj Prasad, Niranjan Baisakh

**Affiliations:** 1 School of Plant, Environmental, and Soil Sciences, Louisiana State University Agricultural Center, Baton Rouge, LA 70803, USA; 2 National Institute of Plant Genome Research, Aruna Asaf Ali Marg, New Delhi 110067, India

## Abstract

ADP-ribosylation factors (ARFs) have been reported to function in diverse physiological and molecular activities. Recent evidences also demonstrate the involvement of ARFs in conferring tolerance to biotic and abiotic stresses in plant species. In the present study, 23 and 25 ARF proteins were identified in C_3_ model- rice and C_4_ model- foxtail millet, respectively. These proteins are classified into four classes (I–IV) based on phylogenetic analysis, with ARFs in classes I–III and ARF-like proteins (ARLs) in class IV. Sequence alignment and domain analysis revealed the presence of conserved and additional motifs, which may contribute to neo- and sub-functionalization of these proteins. Promoter analysis showed the presence of several *cis*-regulatory elements related to stress and hormone response, indicating their role in stress regulatory network. Expression analysis of rice *ARFs* and *ARLs* in different tissues, stresses and abscisic acid treatment highlighted temporal and spatial diversification of gene expression. Five rice cultivars screened for allelic variations in *OsARF* genes showed the presence of allelic polymorphisms in few gene loci. Altogether, the study provides insights on characteristics of *ARF/ARL* genes in rice and foxtail millet, which could be deployed for further functional analysis to extrapolate their precise roles in abiotic stress responses.

ADP-ribosylation factors (ARFs) belong to Ras superfamily of small GTP-binding proteins (GTPases), which regulate broad-spectrum biological processes and molecular functions in all eukaryotes^1^,^2^. These low molecular weight (21–24 kDa) proteins are further classified into ARFs and ARF-like (ARL) proteins based on their functional characteristics and sequence homology^1^. ARFs are highly conserved proteins (>60% sequence identity) sharing similar biological activities, but ARLs are highly divergent (40–60% identity) and function in secretory and other pathways^3^. Unlike other members of Ras superfamily, ARF family lacks the C-terminal isoprenylation and carboxymethylation regions, but possesses an additional nucleotide-sensitive region, an extension at the N-terminus and a covalently attached myristate, which complement each other to constitute a ‘myristoyl switch’^4^. This enables the activation of ARFs by guanine exchange factors (GEFs), which convert inactive GTP-ARF to active, membrane-associated GTP-ARF, whereas GTPase activating proteins (GAPs) revert the active forms to inactive GDP-ARF^1^.

ARFs have been identified in several plant species including *Arabidopsis*, rice, tomato, potato, maize, carrot, wheat, tobacco and barley. In *Arabidopsis*, Regad *et al*.^5^ were the first to isolate *ARF1* from a cDNA library and observed higher accumulation of *ARF1* transcripts in actively-dividing and quiescent cells. Gebbie *et al*.^6^ identified 15 ARF and 4 ARL proteins in the *Arabidopsis* genome*. Arabidopsis* plants with antisense suppression of *ARFA1c* were severely stunted due to decreased cell division, cell expansion and cellulose biosynthesis, which are directly dependent on vesicle trafficking^6^. Zopa and Müller-Rober^7^ isolated *ARFA1* from potato, which was found to be highly expressed in growing tubers of different developmental stages. *ARFA1* under-expression lines of potato showed changes in metabolite synthesis including increased sucrose accumulation and decreased glycoalkaloids level^8^. Further, changes in phenolic compounds enhanced the antioxidant capacity of transgenic plants^8^. Liu *et al*.^9^ reported differential expression of *ARF1* during developmental process of potato tubers. Significantly higher expression of *ARF1* during tuber dormancy breaking suggested its putative involvement in tuber dormancy and sprouting^9^. ARF proteins were shown to be indirectly involved in transformation of the lipid composition in maize^10^, and over-expression of *ZmARF1* in *Arabidopsis* resulted in increased leaf and seed size along with enhanced growth rate^11^. Similarly, over-expression of *ZmARF2* in *Arabidopsis* resulted in cell expansion to produce larger leaves and seeds, and taller plants^12^. *ARFA1* showed higher expression in carrot during somatic embryogenesis, and its over-expression in *E. coli* showed specific binding activity toward GTP^13^. Asakura *et al*.^14^ cloned *ARF1* and *ARF2* from carrot seedlings and showed that *ARF1* was up-regulated in leaf and stem tissues, whereas *ARF2* was highly expressed in root. Kobayashi-Uehara *et al*.^15^ performed cDNA cloning of *ARF1* in wheat and reported relative abundance of the ARF proteins in root and flower tissues. The study showed a correlation of the higher expression of *ARF1* in root and flower tissues with the high level of vesicular transporting activity in these tissues^15^. In barley, *ARF1* was observed to be upregulated during leaf senescence, and functional characterization of this gene revealed its putative involvement in senescence-dependent recycling processes^16^. In rice, *ARF1* showed higher expression in young (2-week-old) seedlings and in seeds at the early developmental stage (0–6 days after pollination)^17^.

Recently, ARF proteins have been shown to play roles in conferring tolerance to biotic as well as abiotic stresses in crop plants. Lee *et al*.^18^ observed differential expression of *ARF1* gene in rice suspension culture cells challenged with *Magnaporthe grisea*. Transgenic tobacco plants over-expressing *OsARF1* showed spontaneous induction of lesion mimics, expression of pathogenesis-related (PR) genes, and higher levels of endogenous salicylic acid and reduced susceptibility to fungal pathogen^18^. In *Arabidopsis*, an ARF-GEF, *AtMIN7* was specifically targeted by *Pseudomonas syringae* virulence factor, HopM1^19^. Coemans *et al*.^20^ reported that loss-of-function of *ARF1* in tobacco severely affected the non-host resistance to *P. cichorii* and partially compromised *N* gene-mediated resistance to TMV^20^.

Higher amount of *ARF1* transcripts was accumulated in response to salinity stress in *Medicago truncatula*^21^, *Spartina alterniflora*^22^ and indica rice cultivars^23^. Joshi *et al*.^24^ showed that overexpression of *ARF1* gene from *S. alterniflora* (*SaARF1*) conferred salt and drought tolerance in rice and *Arabidopsis* transgenic plants^25^. Altogether, these reports signify the importance of *ARF* genes in developmental, physiological and stress responses in different plant species. However, studies on the structure, organization and evolution of *ARF* genes on a genome-wide scale, and a comprehensive expression profiling in response to different stresses are required to delineate their precise roles in stress responses.

Among the plant kingdom, Poaceae family constitutes important grass species of which rice (*Oryza sativa* L.) and foxtail millet (*Setaria italica* L.) are considered as C_3_ and C_4_ model crops, respectively^26^,^27^. Being diverged from a common ancestor around 80 million years ago, rice serves as the staple food for more than half of the global population, whereas foxtail millet is primarily cultivated as a food and fodder crop in arid and semi-arid regions of the world. In rice, a preliminary study identified eight putative *ARF* genes (*OsARF*)^28^; however, no comprehensive analysis of *ARF* gene family members has been reported thus far. Similarly, information on the structure, organization, and evolution of *ARF* genes in foxtail millet are not available yet. The present study was conducted to identify the *ARF/ARL* gene family members in both the crops and to perform a comparative study on their structure, organization, duplication and divergence, and expression patterns in different tissues. Further, expression profiling of *OsARF* genes in response to abiotic stresses and hormone treatment was also performed. Moreover, allelic variations of a few selected genes were studied in five different rice cultivars.

## Results and Discussion

### ARF/ARL proteins of rice and foxtail millet

ADP-ribosylation factors (ARFs) reportedly function in diverse physiological and molecular activities. Recent evidences have also demonstrated their involvement in conferring tolerance to biotic and abiotic stresses in plants. In the present study, a comprehensive genome-wide survey of ADP-ribosylation factors (ARFs) in rice and foxtail millet using keywords, PFAM ID and HMM was performed. The study identified 36 putative ARF proteins in both the genomes. Among the 36 proteins of rice, 13 were found to be alternate transcripts, whereas 11 were splice variants in foxtail millet (Supplementary Table S1). The presence of conserved ADP-ribosylation factor family domain (PF00025) was confirmed by ScanProsite analysis in 23 and 25 putative ARF proteins of rice and foxtail millet, respectively. The number of ARF proteins identified in rice in the present study is higher than eight putative ARFs reported by Zhou *et al*.^28^. The difference is due to the fact that Zhou *et al*.^28^ identified the putative ARFs by performing TBLASTN search against NCBI database using AtARFA1c and OsARFA1d protein sequences as queries. HMMER tool was used to search the protein sequence database of rice and foxtail millet, and the ARF/ARL proteins including the product of alternate transcripts have been identified. Though rice and foxtail millet has 12 and 9 chromosomes, respectively, foxtail millet has a higher number of *ARF/ARL* genes (25) when compared to rice (23). It could be because of the large-scale loss of duplicated genes that might have occurred shortly after whole genome duplication in rice^29^.

**Figure 1:**
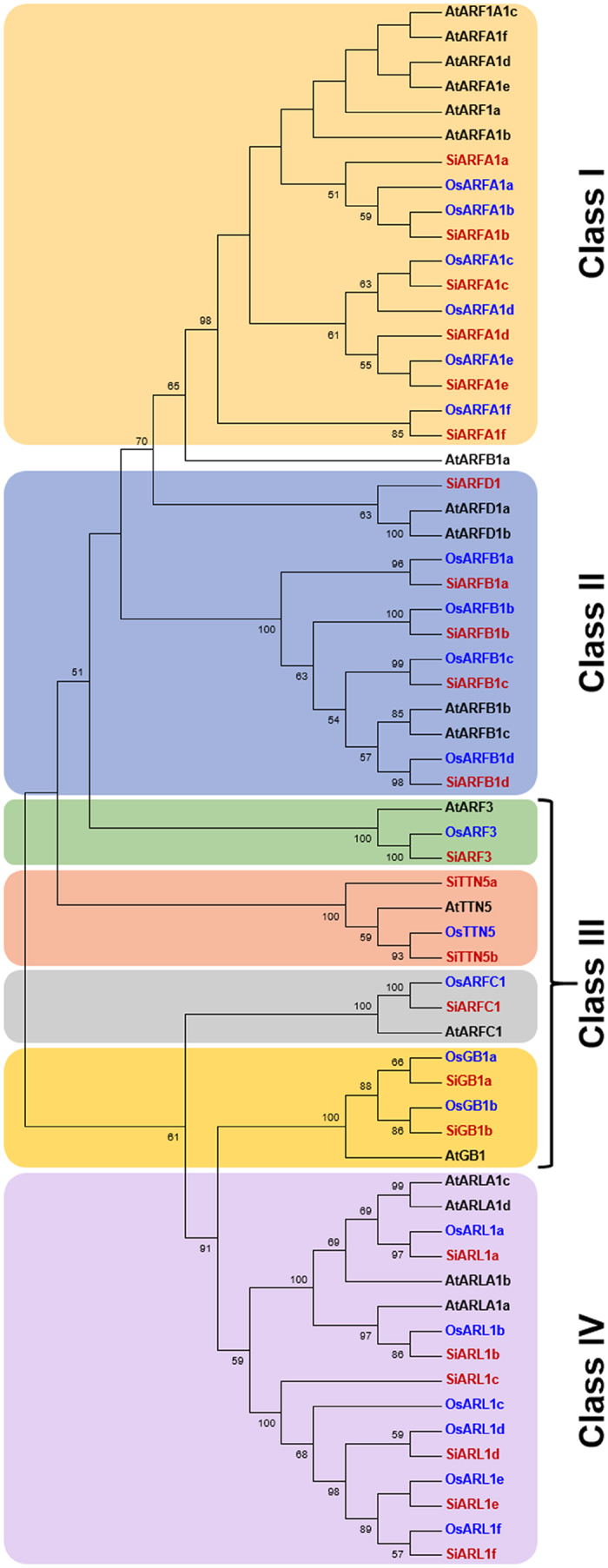
Phylogenetic relationship among ARF/ARL proteins of rice, foxtail millet and *Arabidopsis*. Full-length amino acid sequences of rice (Os), foxtail millet (Si) and *Arabidopsis* (At) were aligned by ClustalW and an unrooted Neighbor-Joining tree was constructed with 1000 bootstrap iterations. The tree classified the proteins into four distinct classes (I–IV), shaded with different colors. The proteins of rice and foxtail millet are represented in blue and red colored fonts, respectively.

An unrooted Neighbor-Joining tree constructed with the deduced amino acid sequences of ARFs and ARLs from rice, foxtail millet, as well as the ARFs and ARLs from *A. thaliana*^6^ classified these proteins into four distinct classes (I to IV), as reported in *Arabidopsis*^6^ (Fig. 1). High bootstrap values at the nodes signified the accuracy of phylogenetic tree where the class I, II and III comprised of ARF proteins, and class IV constituted the members of ARF-like proteins (ARLs). The highly conserved ARFA1 proteins of rice, foxtail millet and *Arabidopsis* formed class I (ARFA1a to ARFA1e), in which AtARFA1 proteins formed a distinct cluster within the class I (Fig. 1). Class II comprised of ARFB1 proteins of the three species along with ARFD1 proteins of foxtail millet and *Arabidopsis*. Both rice and foxtail millet has 4 ARFB1 members (ARFB1a to ARFB1d), and interestingly, rice did not have any ARFD1 protein, while foxtail millet has one member (SiARFD1). The proteins present in class III were classified as ARF3, TTN5, ARFC1 and GB1 proteins based on their homology with respective proteins of *Arabidopsis* (Fig. 1). Rice has two members of GB1 protein (OsGB1a and OsGB1b) and one member each of OsARF3, OsTTN5 and OsARFC1. Foxtail millet has two members each of GB1 (SiGB1a and SiGB1b) and TTN5 (SiTTN5a and SiTTN5b), and one member each of SiARF3 and SiARFC1. Class IV comprised of ARLs, where both rice and foxtail millet have six members (ARL1a to ARL1f), and *Arabidopsis* has four members (Fig. 1). The previous report^28^ grouped the eight putative rice ARF proteins under class I (OsARFA1a to OsARFA1e) and class II (OsARFB1a to OsARFB1c).

**Figure 2:**
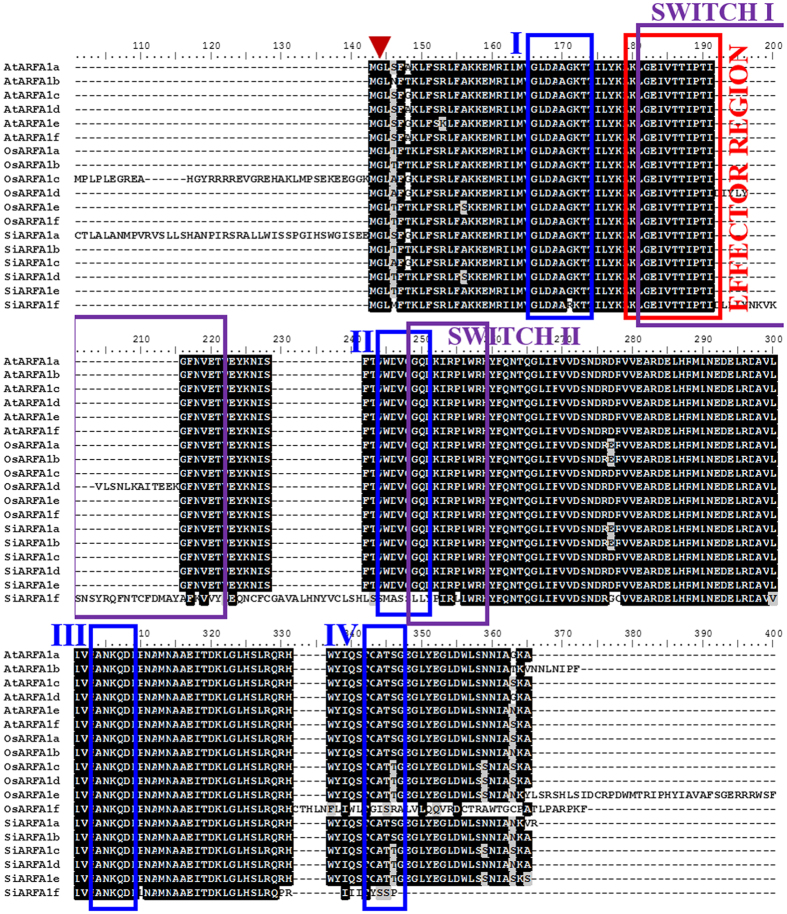
Alignment of class I ARF proteins of rice and foxtail millet with *Arabidopsis* ARFA1 members. Full-length amino acid sequences of class I ARF proteins from rice (Os), foxtail millet (Si) and *Arabidopsis* (At) were aligned using ClustalW. Inverted red triangle indicates the myristoylation site. Blue rectangles represent four GTP-binding domains (I–IV), Purple rectangles represent two GEF Sec7 interaction domains (switch 1 and switch 2), and red rectangle indicates a single GAP interaction domain (effector region). The light or dark shaded backgrounds indicate partial or entirely conserved amino acid residues, respectively.

Sequence alignment and analysis of class I ARF proteins of rice, foxtail millet and *Arabidopsis* revealed the presence of four GTP binding sites^30^, the effector region (GTPase activating site)^31^,^32^,^33^, the switch 1 and switch 2 region binding guanine exchange factors Sec7 domains^3^,^34^ and a potential myristoylation site at Gly-2^6^,^35^,^36^ (Fig. 2). The class I ARFs perform essential physiological functions in plant growth and development^6^. Further, domain analysis of the identified ARF/ARL proteins showed the presence of additional domains other than the conserved ARF domain, PF00025 (Supplementary Table S2). These included signal recognition particle receptor beta subunit (PF09439), Ras of Complex (Roc) domain of DAPkinase (PF08477), Ras family (PF00071), Gtr1/RagA G protein conserved region (PF04670) and 50S ribosome-binding GTPase (PF01926). Among the class I proteins, OsARFA1d and SiARFA1f had two ARF domains; however, SiARFA1f was devoid of any other domains except two ARF domains. In case of the class II proteins, OsARFB1b and SiARFB1b contained all six domains, and rest of the members lacked 50S ribosome-binding GTPase domain. Similarly, all the members of class III proteins of foxtail millet and rice except OsTTN5 possess all six domains. Of note, SiTTN5b was observed to have two ARF domains (Supplementary Table S2). The domain composition of OsARLs and SiARLs was similar to ARL1a and ARL1b proteins of both rice and foxtail millet as they possess all six domains, whereas ARL1c lacked Ras family (PF00071) and Gtr1/RagA G protein conserved region (PF04670). Further, ARL1d, ARL1e and ARL1f proteins of rice and foxtail millet did not have the Gtr1/RagA G protein conserved region (Supplementary Table S2). However, the binding domains as predicted in ARF/ARL proteins may not necessarily be utilized by the binding proteins, and therefore, their interactions need experimental validation.

Among the 15 OsARF proteins (class I to III), OsARFA1c was found to be the largest protein with 304 amino acids (34.7 kDa), whereas OsARFA1a (20.6 kDa) and OsARFA1b were the shortest with 181 amino acids each (Supplementary Table S1). On the other hand, SiARFA1a of foxtail millet was the largest protein with 324 amino acids (35.6 kDa) and the shortest was SiTTN5b with 159 amino acids (18.1 kDa) (Supplementary Table S1). Among the OsARLs, OsARL1c was the largest protein with 194 amino acids, but OsARL1f had highest molecular weight of 22 kDa. Among the SiARLs, SiARL1b was the largest protein with 278 amino acids (30.2 kDa). The isoelectric pH (pI) of OsARFs ranged from 5.4 (OsARF3) to 9.2 (OsARFA1f) and OsARLs ranged from 5.9 (OsARL1c) to 9.1 (OsARL1a). In case of SiARFs, the pI ranged from 5.2 (SiARF3) to 10 (SiARFA1a) and among the SiARLs, the values ranged from 9.3 (SiARL1b) to 6 (SiARL1c) (Supplementary Table S1). The instability index of ARF/ARL proteins showed that OsARFA1c, OsARFA1e, OsARFB1b and OsGB1b of OsARF family, and SiGB1b, SiARFD1 and SiARFA1a of SiARF family were unstable. All the members of OsARLs and SiARLs except SiARL1b were predicted to be stable (Supplementary Table S1). Large variations in the protein features, such as the length, molecular mass, pI and instability index of ARF/ARL proteins of rice and foxtail millet, attribute to the functional divergence of ARF/ARL family members.

### Gene structure and chromosomal location of ARF/ARL protein encoding genes

The genes showed variations in their size and the number of introns and exons (Supplementary Table S1). Among the *OsARF* genes, *OsTTN5* was the longest (5.6 kb) and the shortest was *OsARFB1a* (0.9 kb). In case of *OsARL* genes, *OsARL1d* and *OsARL1e* were the longest (5.1 kb) and the shortest (2.3 kb) genes, respectively. Among the *SiARF* genes, *SiARF3* was the longest (6.6 kb), whereas *SiARFB1a* was the shortest gene (1 kb). *SiARL1d* and *SiARL1c* were the longest and the shortest genes among *SiARLs* with the size of 3.8 kb and 8.5 kb, respectively.

Comparison of the intron-exon organization of ARF/ARL protein-encoding genes between the members of rice and foxtail millet showed that there was no similarity in the structures of class I protein encoding genes between rice and foxtail millet, whereas most of the sister-gene pairs belonging to other three classes had similar intron-exon organization (Supplementary Fig. S1). Though these genes have similar number of introns and exons, the lengths of the introns and exons as well as the gene lengths were generally different. Class I protein encoding genes of rice had a maximum of five introns (*OsARFA1c*, *OsARFA1d*, *OsARFA1e*, and *OsARFA1f*) and a minimum of four introns (*OsARFA1a* and *OsARFA1b*), whereas in foxtail millet, *SiARFA1a* possessed a maximum of six introns, *SiARFA1b* thru *SiARFA1e* had 4 introns and *SiARFA1f* had a minimum of three introns (Supplementary Fig. S1). The genomic structural organizations of the *OsARF* genes are in congruence with the findings reported by Zhou *et al*.^28^. However, there was little similarity in gene structure of *ARFs* and *ARLs* of rice and foxtail millet compared to *AtARF/ARL* genes reported by Gebbie *et al*.^6^.

**Figure 3:**
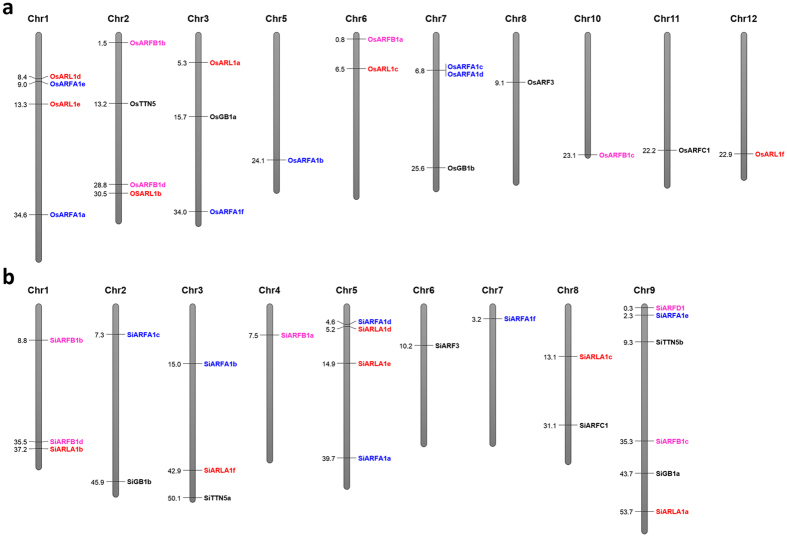
Physical map of rice and foxtail millet showing the chromosomal location of *ARF/ARL* genes. *ARF/ARL* genes were mapped onto the chromosomes of (**a**) rice and (**b**) foxtail millet to construct the physical map. The vertical bars represent individual chromosomes with numbers on left indicating physical position (in Mb). The different classes of *ARF/ARL* genes are shown in different font colors.

The *ARF/ARL* genes identified in *Arabidopsis*^6^ and rice^28^ were not physically mapped onto the respective genomes. Physical mapping of the *ARF/ARL* genes of rice and foxtail millet to their respective genomes showed that *OsARF* genes were present on all the chromosomes of rice except chromosome 4, 9 and 12, with a maximum of three genes each on chromosome 2 and 7, and a minimum of one gene each on chromosome 5, 6, 8, 10 and 11 (Fig. 3). *OsARL* genes were distributed over chromosomes 1, 2, 3, 6 and 12, with a maximum of two genes on chromosome 1. On the contrary, *SiARF* genes were spread over all nine chromosomes of foxtail millet, with a maximum of five genes on chromosome 9, and a minimum of one gene each on chromosome 4, 6, 7 and 8. Chromosome 1, 3, 5, 8 and 9 harbored *SiARL* genes and of these, chromosome 5 had a maximum of two genes (Fig. 3). Altogether, the results revealed an uneven distribution of *ARF/ARL* genes on rice and foxtail millet chromosomes. This is in agreement to the distribution of genes belonging to other gene families including Sodium/Calcium exchanger gene family in rice and *Arabidopsis*^37^, and MYB^38^, C_2_H_2_-zinc finger^39^ and WRKY^40^ gene families in foxtail millet. The physical map constructed in the present study will be useful in identifying candidate genes for further characterization and map-based cloning of those genes. Further, it will also assist in the construction of comparative maps between related genomes.

Genome duplication plays a significant role in plant diversification, and duplication of genes during the course of evolution results in the presence of multiple copies of genes belonging to the same family. In order to understand the role of duplication, gene duplication analysis was performed to identify *ARF/ARL* paralogs of rice and foxtail millet, which showed that these genes did not undergo tandem or segmental duplication in the respective genomes. Similar observation has been reported on RNA-dependent RNA polymerase gene family in rice^41^ and foxtail millet^42^, in which the role of duplication on expansion of this gene family could not be deduced. However, further in-depth study is required to deduce and confirm the paralogous relationship among the *ARF/ARL* genes of rice and foxtail millet.

### *Cis*-regulatory elements in promoters of *ARF/ARL* family genes

Analyzing the *cis*-regulatory elements in the promoter region of *ARF/ARL* family genes is important for delineating their function and regulation. Scanning the upstream sequences (2 kb) of each gene identified a total of 238 and 228 *cis*-regulatory elements present in the promoter region of *ARF/ARL* family genes of rice and foxtail millet, respectively (Supplementary Table S3). In both crops, a few *cis*-elements were found in the promoter region of all the genes and a few elements were present only in the promoter sequence of any one gene. In case of rice, elements showing response to dehydration (ACGTATERD1), cytokinin (ARR1AT), copper stress (CURECORECR), light (EBOXBNNAPA, GT1CONSENSUS and SORLIP1AT), salt and biotic stress (GT1GMSCAM4), cold stress (MYCCONSENSUSAT) and wounding (WBOXNTERF3) were present in promoter region of all the *OsARF* and *OsARL* genes. In addition, a few common elements including CAATBOX1 (element in enhancer region of the promoter), WRKY71OS (WRKY transcription factor binding), DOFCOREZM (Dof proteins binding) and MYBCORE (MYB transcription factor binding) were present in all the rice *ARF/ARL* genes. The elements that were unique to any one member of *ARF/ARL* family in rice included ABREA2HVA1 (abscisic acid- and water stress- responsive element in *OsARFB1d*), DRE1COREZMRAB17 (dehydration responsive element in *OsARFA1b*), HBOXCONSENSUSPVCHS (elicitor responsive element in *OsGB1a*), LTREATLTI78 (low temperature responsive element in *OsARFB1a*), O2F2BE2S1 (opaque-2 recognition site in *OsARFC1*), PIATGAPB (light responsive element in *OsGB1a*), SBOXATRBCS (drought responsive element in *OsTTN5*), TELOBOXATEEF1AA1 (telomere motif in *OsARL1e*) and coupling elements for the G box (CACGCAATGMGH3 in *OsARFA1f* and CARGATCONSENSUS in *OsARFC1*) (Supplementary Table S3).

Similar to rice, the promoter region of all the *SiARF* and *SiARL* genes possessed ACGTATERD1 (dehydration responsive), ARR1AT (cytokinin responsive), CURECORECR (copper stress responsive), EBOXBNNAPA, GT1CONSENSUS and SORLIP1AT (light responsive), MYCCONSENSUSAT (cold responsive), and WBOXNTERF3 (wound responsive) elements, in addition to the commonly found DOFCOREZM, MYBCORE and WRKY71OS elements (Supplementary Table S4). Moreover, all the *SiARF* and *SiARL* genes were observed to possess nodule-specific element (NODCON2GM), sugar responsive element (WBOXHVISO1) and carbon dioxide responsive element (EECCRCAH1). The *cis*-regulatory elements present only in any one member of *SiARFs* and *SiARLs* included ABA-responsive elements (ABREATCONSENSUS in *SiARLA1f*, ABREATRD22 in *SiARLA1d* and ABREMOTIFAOSOSEM in *SiTTN5a*), antioxidant response element (ARE1 in *SiTTN5b*), auxin response element (AUXRETGA2GMGH3 in *SiARLA1f*), drought, low-temperature and high-salt stress responsive element (DREDR1ATRD29AB in *SiGB1a*), gibberellin responsive element (GARE2OSREP1 in *SiARFB1a*), and sucrose responsive element (SURE1STPAT21 in *SiARFA1c*) (Supplementary Table S4). The analysis provides a fundamental understanding on the transcriptional control of *ARF/ARL* genes in stress response and developmental processes, and this would enable further functional characterization of promoters for understanding the regulation of gene expression.

### Gene ontology annotation of *OsARF/OsARL* family members

Gene ontology (GO) annotation was performed for *ARF/ARL* family members of rice to identify biological process in which these proteins are involved, their molecular functions and cellular component (Supplementary Fig. S2). The analysis revealed that 21 proteins were involved in small GTPase-mediated signal transduction, 19 proteins in intracellular protein transport, 17 in protein ADP-ribosylation, and 15 in vesicle-mediated transport. Six proteins were also predicted to participate in the cellulose biosynthesis process (Supplementary Fig. S2). In accordance to their biological processes, the molecular functions of the *ARF/ARL* proteins were identified as GTP binding (21 proteins), with transporter activity (18 proteins) and calcium ion binding (9 proteins). Further, cellular component analysis showed that the majority of proteins were localized in the mitochondrion (12 proteins), followed by plasma membrane (7 proteins) and golgi apparatus (6 proteins) (Supplementary Fig. S2). The GO data will be useful in finding functional similarity among differentially expressed genes and analyzing and constructing co-expression networks of the gene families during developmental or stress phase of the plants.

### Tissue-specific expression profiles of *ARF/ARL* family genes

**Figure 4:**
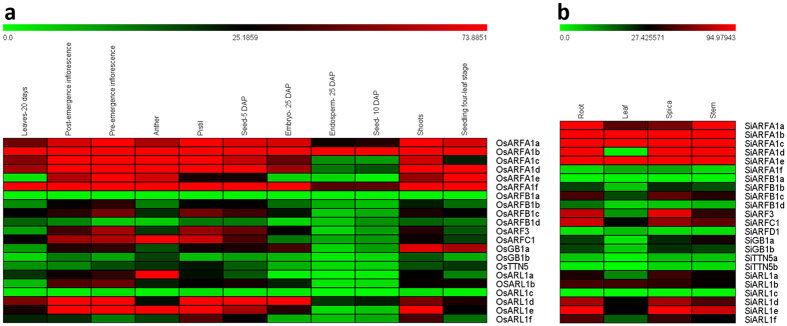
RNA-seq based expression profiles of *ARF/ARL* genes in different tissues of rice and foxtail millet. Heatmap was generated for (**a**) rice and (**b**) foxtail millet based on FPKM values derived from tissue-specific transcriptome data. The scale bar at the top represents relative expression values.

The publicly available RNA-seq libraries of rice and foxtail millet were processed for analyzing the expression profiles of *ARF/ARL* family genes. Heatmap constructed from normalized FPKM values of RNA-seq data from eleven tissues of rice (leaf, post- and pre-emergence inflorescence, anther, pistil, seeds at 5 and 10 DAP, embryo, endosperm, shoot, and seedling at four leaf stage) and four tissues of foxtail millet (root, leaf, spikelet and stem) showed differential pattern of gene expression in the tissues (Fig. 4; Fig. S3). A few orthologous genes, such as the up-regulation of *ARFA1b* and down-regulation of *ARFB1a* and *ARL1c*, displayed similar expression pattern in rice and foxtail millet.. Most of the genes were not expressed in endosperm and seed of rice, and higher expression of *OsARFC1* and *OsARL1a* in anther and *OsGB1a* in shoots was observed. In case of foxtail millet, all the class I protein encoding genes except *SiARFA1f* showed higher-expression in all four tissues. On the other hand, *SiARFA1d* did not show any expression in leaf.

**Figure 5:**
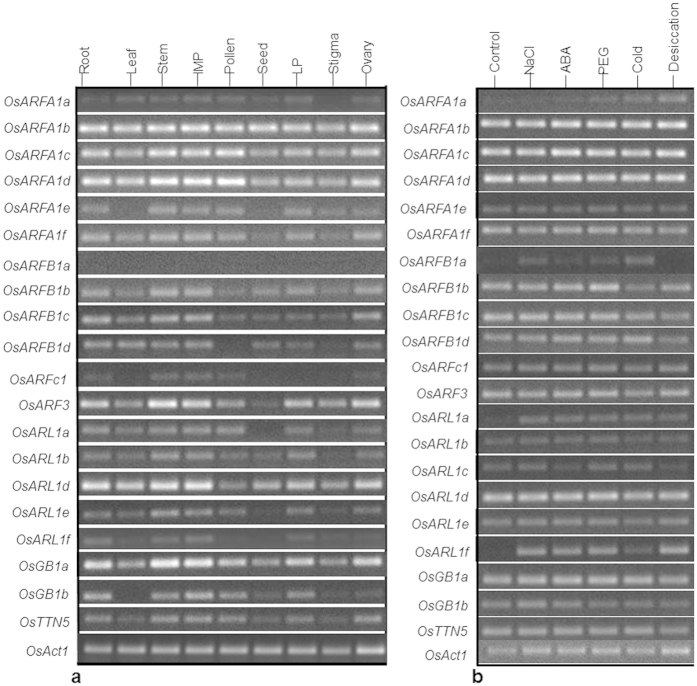
Expression profiles of *OsARF* and *OsARL* genes in different tissues, stresses and hormone treatment. Semi-quantitative RT-PCR derived expression pattern of *OsARF* and *OsARL* genes (**a**) in different tissues of rice namely, root, leaf, stem, immature panicle (IMP), pollen, seed, lemma and palea (LP), stigma and ovary, and (**b**) under salinity (150 mM NaCl), dehydration (20% PEG2250), cold (4 °C), desiccation (resting seedlings on filter paper) and abscisic acid (50 μM) treatments for 24 h in three week-old rice seedlings. *OsAct1* was used as an internal reference control.

The expression pattern of *OsARF* and *OsARL* genes in different tissues namely root, leaf, stem, immature panicle (IMP), pollen, seed, lemma and palea (LP), stigma, and ovary by semi-quantitative RT-PCR analysis showed a clear spatial expression pattern of predominant genes in tissues of *O. sativa* japonica ‘Nipponbare’ (Fig. 5a). Among the class I protein encoding genes, *OsARFA1b*, *OsARFA1c*, *OsARFA1d* and *OsARFA1f* showed expression in all tissues at varying level, whereas *OsARFA1a* did not have detectable mRNA accumulation in stigma and *OsARFA1e* transcript was not accumulated in leaf and seed. Among the class II protein encoding genes, *OsARFB1a* did not show any expression in all tissues, but expression of *OsARFB1b*, *OsARFB1c* and *OsARFB1d* was significant in root, leaf, stem, immature panicle, pollen and ovary. All the class III protein encoding genes showed high levels of expression in stem and immature panicle. *OsARF3* and *OsGB1a* showed similar expression pattern, and *OsARFC1* transcript was detected only in root, stem, immature panicle, pollen and ovary. A relatively lesser level of expression of *OsGB1b* in comparison to *OsGB1a* was observed in all tissues, and it was not expressed in leaf (Fig. 5a). Among the *OsARL* members, *OsARL1d* showed significantly higher expression compared to others, whereas *OsARL1f* was expressed in very low levels in all tissues with no or less expression in pollen and seed. The expression profile suggested the ubiquitous roles of genes which showed no significant difference in expression, and tissue-specific biological and molecular roles of genes which are highly expressed in particular tissues. Previously, Zhou *et al*.^28^ have analyzed the expression pattern of eight *OsARF* genes in ten tissues namely young and mature leaves, sheath, stem, root, flower, glume, endosperm, caryopsis and cultured cells. The study reported higher accumulation of all *OsARF* transcripts except *OsARFA1a* in root and reproductive organs. The tissue-specific expression pattern of *OsARF/ARLs* identified in the present study conforms to Zhou *et al*.^28^, which suggested the involvement of *ARF/ARL* family genes in plant growth and developmental processes.

### Orthologous relationships of *ARF/ARL* family genes among poaceae members

Sequence-based orthologous relationships derived between the genomes of rice, foxtail millet, sorghum and maize using *ARF/ARL* family genes (>80% identity) showed that the *ARFA1b*, *ARFA1c* and *ARFA1e* genes from class I; *ARFB1b*, *ARFB1c* and *ARFB1d* from class II; *GB1* and *TTN5* from class III; and *ARL1a*, *ARL1b*, *ARL1d* and *ARL1e* from class IV are conserved in all four genomes (Supplementary Tables S5,S6). The orthologous relationship between *ARF/ARL* genes of rice and other poaceae members revealed higher gene-based synteny between rice and sorghum (18 genes), followed by foxtail millet and maize (16 genes) (Supplementary Table S5). Few class I protein encoding genes, such as *OsARFA1a*, *OsARFA1d* and *OsARFA1f* did not show any orthologous relationship with sorghum, foxtail millet and maize. Foxtail millet *ARF/ARL* family genes showed maximum synteny with sorghum (16 genes), followed by maize (13 genes) (Supplementary Table S6). This extensive gene-level synteny shared among rice, foxtail millet, sorghum and maize supported their close evolutionary relationships^43^,^44^; however, it also suggested the role of chromosomal rearrangements such as duplication, inversion and deletion in shaping the distribution and organization of ARF/ARL genes in these genomes. Though the occurrence of tandem and segmental duplications has not been implicated in the expansion of *ARF/ARL* gene family in these genomes, dispersed gene duplication, which occurs through either DNA or RNA based transposition mechanisms^45^,^46^,^47^, might have played a role in evolving paralogous genes and imparting sub-, neo- and non-functionalization to these genes^48^,^49^.

Based on these results, the effect of Darwinian selection in the divergence of *ARF/ARL* genes was analyzed by calculating the substitution ratios (Ka/Ks) of the orthologous gene-pairs. The mean Ka/Ks of these gene pairs were found to be <1, which indicated that these gene-pairs have diverged under the influence of strong positive selection. Further the time of divergence of orthologous gene-pairs between rice and -sorghum, -foxtail millet, and –maize was, on an average, calculated to be 48.5 million years ago (mya), which coincided with the divergence of these crops from a common ancestor around 50 mya^50^,^51^ (Supplementary Table S5). Similarly, the time of divergence of orthologous gene-pairs between foxtail millet and -sorghum, and -maize was calculated as ~28 mya, which is in agreement with the divergence of foxtail millet from sorghum and maize at ~27 mya^43^ (Supplementary Table S6). Understanding the orthologous relationships among the ARF/ARL genes will be useful in the construction of comparative genome maps for identifying candidate genes for functional analyses.

### Expression profiles of *OsARF* and *OsARL* genes in response to stress and ABA treatment

The expression analysis of *OsARF* and *OsARL* genes in response to different stresses (NaCl, PEG, cold and desiccation) and ABA treatment in revealed differential expression of a few genes under the treatments (Fig. 5b). Of note, many genes showed class-specific similar expression patterns. Among the class I protein encoding genes, the levels of *OsARFA1a* showed an increased mRNA accumulation under stress, especially under PEG, cold and desiccation in comparison with the control. However, other members of this class did not show any difference in their expression as compared to control. Among the class II protein encoding genes, *OsARFB1a* was expressed only under stress with the exception of desiccation. *OsARFB1b* showed higher expression in response to treatment with ABA and PEG and downregulation under cold (Fig. 5b). On the other hand, *OsARFB1c* and *OsARFB1d* were down-regulated during desiccation. Class III protein encoding genes did not show any significant differential expression patterns in response to stress and ABA treatment. However, *OsGB1b* levels were relatively higher under NaCl, PEG and ABA treatments. In the case of *OsARL* genes, *OsARL1a* was upregulated under all stress and ABA treatments compared to the control, and other genes, such as *OsARL1b*, *OsARL1d* and *OsARL1e* did not show any change in their expression patterns as compared to the control. *OsARL1c* was observed to be down-regulated during ABA and desiccation. *OsARL1f* showed significant upregulation under all stresses, except cold and ABA treatments (Fig. 5b). The present study is, to our knowledge, the first to report the expression pattern of *OsARF/ARL* genes in response to different abiotic stresses and hormone treatments, although stress-induced upregulation of *ARFA1a* has previously been reported by Joshi *et al*.^25^. The authors have shown that transgenic rice and *Arabidopsis* lines overexpressing *ARFA1a* gene from the halophyte *S. alterniflora* (smooth cordgrass) exhibited enhanced tolerance to salt and drought stress than wild-type plants. The expression profiles of *OsARF/ARL* genes could be correlated with the *cis*-regulatory elements present in the promoter regions of respective genes. The genes *OsARFA1a, OsARFB1a, OsARFB1b, OsGB1b, OsARL1a* and *OsARL1f* that were up-regulated during dehydration and salinity stress have one or more response to dehydration stress *cis*-motifs, such as ABRELATERD1, ACGTATERD1 and MYCATRD22 in their promoter regions^52^,^53^. Similarly, the genes *OsARFA1a, OsARFB1a* and *OsARL1a* that exhibited higher expression during cold stress possess a cold stress responsive CACGT motif 52 in their promoter. The genes *OsARFB1b, OsGB1b* and *OsARL1a* that were highly accumulated under ABA treatment possess both MYCCONSENSUSAT and MYCATRD22 cis-motifs, suggesting their putative roles in ABA-dependent stress response^53^. Though the study suggested an interaction of *cis*-elements and transcription factors for differential/condition-specific gene expression in response to abiotic stresses and hormone treatment^54^,^55^, further characterization of the *cis*-elements through promoter deletion-reporter gene fusion experimentations will validate promoter regulated expression of the genes.

### Allelic variation of *OsARF* genes

Allelic polymorphism in seven *OsARF* genes, namely *OsARFA1a, OsARFA1b, OsARFA1c, OsARFA1e, OsARFB1b, OsARFB1c* and *OsARFB1d*, were examined in five rice cultivars namely ‘Nipponbare’, ‘IR64’ (drought sensitive) and ‘Vandana’, ‘N22’, and ‘Azucena’ (drought tolerant). Diverse mutations, including single nucleotide polymorphisms (SNPs), and insertions and deletions (InDels), were observed in the amplified locus of these genes (Supplementary Fig. S4). For example, a non-synonymous transition of T to C at the 76^th^ base (phenylalanine to alanine) of *OsARFB1d* locus was detected in drought tolerant cultivars ‘Vandana’ and ‘N22’. In addition, a few base changes and deletion mutations were also present in the upstream sequences of *OsARFB1d* in these two cultivars. The upstream sequence of *OsARFA1a* contained a deletion of 27 bases in ‘Vandana’ and ‘N22’. Four distinct allelic variations specific to these two cultivars were also detected in *OsARFA1a* locus. These data suggested the existence of potential allelic variations in *ARF/ARL* genes of stress tolerant and susceptible cultivars, which need to be investigated further for validation, and be extrapolated for development of allele-specific functional markers to expedite marker-assisted breeding of elite cultivars with abiotic stress tolerance.

## Conclusions

The present study identified 23 and 25 ARF genes present in rice and foxtail millet, respectively, which were diverged from a common ancestor around 80 mya. Although rice has 12 chromosomes compared to foxtail millet (9 chromosomes), the number of ARF genes is more in foxtail millet than rice. Similarly, the structure and protein properties of these genes varied drastically between the two species except for Class I proteins, which were apparently evolutionarily conserved. Further, sequence and domain analysis indicated the presence of several additional domains in addition to conserved ARF domain, which suggested the neo- and sub-functionalization of ARF proteins. The comparative mapping analysis provided clues on evolutionary perspective of *ARF/ARL* genes. ARF genes expressed in a specific tissues and under a particular stress need to be studied in detail, which could provide further clues to identification of tissue-specific and/or stress-inducible promoters. Furthermore, validation of SNPs and InDels identified in specific ARF genes in larger collection of stress-tolerant cultivars could lead to the development of allele-specific markers for drought resistance. Taken together, the present study provided comprehensive insights into the structure, organization, evolution and expression profiles of *ARF/ARL* gene family in rice and foxtail millet. Although ARFs have been implicated in biotic stresses of plants, their role in abiotic stress responses has not been thoroughly investigated. The present study provides clues to identifying candidate ARF genes for further functional analysis to delineate their precise role in abiotic stress response.

## Materials and Methods

### Identification of ARF proteins in rice and foxtail millet

Three independent approaches were used to retrieve the ARF proteins from rice and foxtail millet. First, the protein sequences of ARFs and ARLs of *A. thaliana* reported by Gebbie *et al*.^6^ were retrieved and an HMM profile was created (Finn *et al*. 2011). The HMM profile was searched against the protein sequences of rice (retrieved from RGAP release 7: http://rice.plantbiology.msu.edu/) and foxtail millet (retrieved from Phytozome v10.3: http://phytozome.jgi.doe.gov/) using HMMER v3.1b2 with default parameters^56^. Second, HMM profile of ARF proteins downloaded from PFAM (ID: PF00025; http://pfam.xfam.org/) was BLAST searched against the protein sequences of rice and foxtail millet using HMMER v3.1b2 with default parameters. Third, keyword search was performed using the PFAM ID (PF00025) in RGAP and Phytozome for identifying the putative ARF proteins present in rice and foxtail millet, respectively. The resultant gene IDs obtained from all three searches were pooled together and redundant IDs were removed. The unique proteins were further confirmed for the presence of ARF domain using ScanProsite analysis^57^, and subsequently, the sequence information, alternate transcripts, chromosomal location and gene orientation were retrieved for each ID from Phytozome.

### Domain analysis, protein properties, sequence alignment and phylogenetic classification

The protein sequences of identified ARFs were analyzed using HMMSCAN (http://www.ebi.ac.uk/Tools/hmmer/search/hmmscan) to identify different domains present in these proteins. ExPASy - ProtParam tool (http://web.expasy.org/protparam/) was used to compute the properties including molecular weight, theoretical pI and instability index of the proteins. Multiple sequence alignment of ARF proteins of rice and foxtail millet with ARF and ARL proteins of *Arabidopsis* was performed using ClustalW module of BioEdit v7.2.5 (http://www.mbio.ncsu.edu/BioEdit/bioedit.html) with default parameters. The alignment file was then imported into MEGA v6^58^ to construct unrooted Neighbor-Joining phylogeny tree with 1000 bootstrap iterations. The tree constructed with AtARF and AtARL proteins assisted in the identification of ARFs and ARLs in rice (OsARFs and OsARLs) and foxtail millet (SiARFs and SiARLs).

### Analysis of gene structure, chromosomal location and duplication

The CDS and genomic sequences of ARFs and ARLs of rice and foxtail millet were analyzed to predict the gene structure using Gene Structure Display Server v2^59^. The chromosomal coordinates of the identified genes were then used as input for MapChart v2.2^60^ to map the genes on the genome of rice and foxtail millet in ascending order of physical position (Mb), from the short arm telomere to the long arm telomere. The physical map was manually examined to identify tandem duplications as they were characterized as adjacent genes of same sub-family located within 10 predicted genes apart or within 30 kb of each other^61^. For identifying segmental duplication, ~10 kb upstream and downstream sequences of each gene was retrieved and BLASTN searched against the respective genome in Phytozome at1e-10. The significant matches were further verified using Multiple Collinearity Scan toolkit (MCScanX)^62^ following the procedure described by Lee *et al*.^63^.

### Promoter sequence analysis and gene ontology annotation

Upstream sequences (~2 kb) of identified genes were retrieved from Phytozome and the data was analyzed using Plant Promoter Analysis Navigator^64^ (http://PlantPAN2.itps.ncku.edu.tw) for identification of *cis*-regulatory elements in the promoters. The protein sequences of OsARFs and OsARLs were imported into Blast2GO^65^ and cloud-based BLASTN was performed against *Oryza sativa* protein database (taxa: 4530), followed by mapping, annotation, InterProScan and visualization of biological process, molecular functions and cellular components using default parameters.

### Expression profiling using tissue-specific transcriptome data

Raw RNA-seq data of root (SRX128223), stem (SRX128225), leaf (SRX128224) and spica (SRX128226) of foxtail millet were retrieved from European Nucleotide Archive (http://www.ebi.ac.uk/ena). Filtering of the reads were done using NGS toolkit, and mapping of the filtered reads on foxtail millet genome was performed using CLC Genomics Workbench v4.7.1 and normalized using the FPKM method. RNA-seq FPKM expression values for eleven tissues of rice, namely leaf, post- and pre-emergence inflorescence, anther, pistil, seed at 5 and 10 DAP, embryo, endosperm, shoot, and seedling at four leaf stage were retrieved from the Rice Genome Annotation Project database (http://rice.plantbiology.msu.edu/index.shtml).The heatmaps were generated using MultiExperiment Viewer (MeV) v4.9^66^.

### Derivation of orthologous relationships among sequenced grass genomes

The sequences of ARF and ARL proteins of rice and foxtail millet were BLASTN searched against the protein database of sorghum and maize and hits with >80% sequence similarity were used to perform reciprocal BLAST to ascertain the unique relationships between the orthologous proteins. Similar analysis was performed between the members of rice and foxtail to derive the orthologous relationships. The results were further confirmed using OrthoMCL v5^67^ (http://www.orthomcl.org/). The synonymous (Ks) and non-synonymous (Ka) substitution rates for the orthologous pairs were calculated using PAL2NAL sever^68^ and the time of divergence (T) was calculated using the formula T = Ks/(2 × 6.5 × 10^−9^) × 10^−6^ million years ago (mya), based on the rate of 6.5 × 10^−9^ substitutions per site per year for monocots^69^.

### Plant materials and treatments

Seeds of rice cultivars, ‘Nipponbare’, ‘Vandana’, ‘N22’, ‘Azucena’ and ‘IR64’ were grown in greenhouse as described in Baisakh *et al*.^24^. For stress treatments, 3-week-old ‘Nipponbare’ seedlings were subjected to salinity (150 mM NaCl) and dehydration (20% PEG2250) under hydroponics^24^, cold (4 °C), desiccation (resting seedlings on filter paper overnight) and abscisic acid (50 μM) treatments. Leaf tissues were collected after 24 hours, immediately frozen in liquid nitrogen and stored at −80 °C until RNA isolation. For tissue-specific expression profiling, root, leaf, stem, immature panicle (IMP), pollen, seed, lemma and palea (LP), stigma and ovary tissues were collected and snap-frozen in liquid nitrogen and stored at −80 °C till RNA isolation. For DNA isolation, three week-old seedlings of all the five cultivars were collected, frozen in liquid nitrogen and stored at −80 °C.

### Semi-quantitative reverse transcription PCR

Primers were designed from 3′UTR of each *OsARF/ARL* transcript sequence using Primer3 v0.4 (http://bioinfo.ut.ee/primer3-0.4.0/) (Supplementary Table S7). The total RNA was isolated using RNeasy plant mini kit (Qiagen, USA) following manufacturer’s instruction, and the quality and quantity was determined using 1.2% formamide denaturing agarose gel electrophoresis and ND-1000 spectrophotometer (Nanodrop Technologies, Wilmington, DE). First strand cDNA synthesis was performed with 1 μg RNA using iScript^TM^ cDNA synthesis kit (Bio-Rad, Hercules, CA). Semi-quantitative and quantitative real-time PCR were performed in three biological replicates following the procedure described earlier^25^, using *OsAct1* gene as the internal control. The reaction mixtures, experimental conditions, melt curve analysis, and agarose gel electrophoresis were performed following Baisakh *et al*.^24^.

### DNA isolation, PCR amplification and sequencing

Genomic DNA was isolated from all five rice cultivars following the miniCTAB protocol as described earlier^24^. One hundred ng of DNA was amplified with gene-specific primers (http://bioinfo.ut.ee/primer3-0.4.0/) (Supplementary Table S7). PCR reaction set up and thermal profile were same as described earlier^24^. PCR products were cloned into pGEM T-easy vector (Promega, Madison, WI) following manufacturer’s instruction. Insert-containing plasmids were sequenced using an in-house ABI 3130 xL DNA analyzer (Applied Biosystems Inc., Carlsbad, CA). The sequences were filtered for removing vector sequence using VecScreen (http://www.ncbi.nlm.nih.gov/tools/vecscreen/) and aligned using BioEdit v7.2.5.

## Additional Information

**How to cite this article**: Muthamilarasan, M. *et al*. Structure, organization and evolution of ADP-ribosylation factors in rice and foxtail millet, and their expression in rice. *Sci. Rep*. **6**, 24008; doi: 10.1038/srep24008 (2016).

## Supplementary Material

Supplementary Information

Supplementary Dataset 1

Supplementary Dataset 2

Supplementary Dataset 3

Supplementary Dataset 4

Supplementary Dataset 5

Supplementary Dataset 6

Supplementary Dataset 7
