# Supplementary material for: Structure, organization and evolution of ADP-ribosylation factors in rice and foxtail millet, and their expression in rice
Source: Sci Rep. 2016 Apr 21;6:24008. doi: 10.1038/srep24008 (PMC4838888; doi:10.1038/srep24008)

Supplementary Information

Structure, organization and evolution of ADP-ribosylation factors in rice and foxtail millet, and their expression in rice

Mehanathan Muthamilarasan[1,2], Venkata R Mangu[1], Hana Zandkarimi[1], Manoj Prasad[2], Niranjan Baisakh[1,*]

Supplementary Fig. S1. Gene structure of ARF/ARL family genes in rice and foxtail millet. The exons and introns are presented by boxes and lines, respectively.

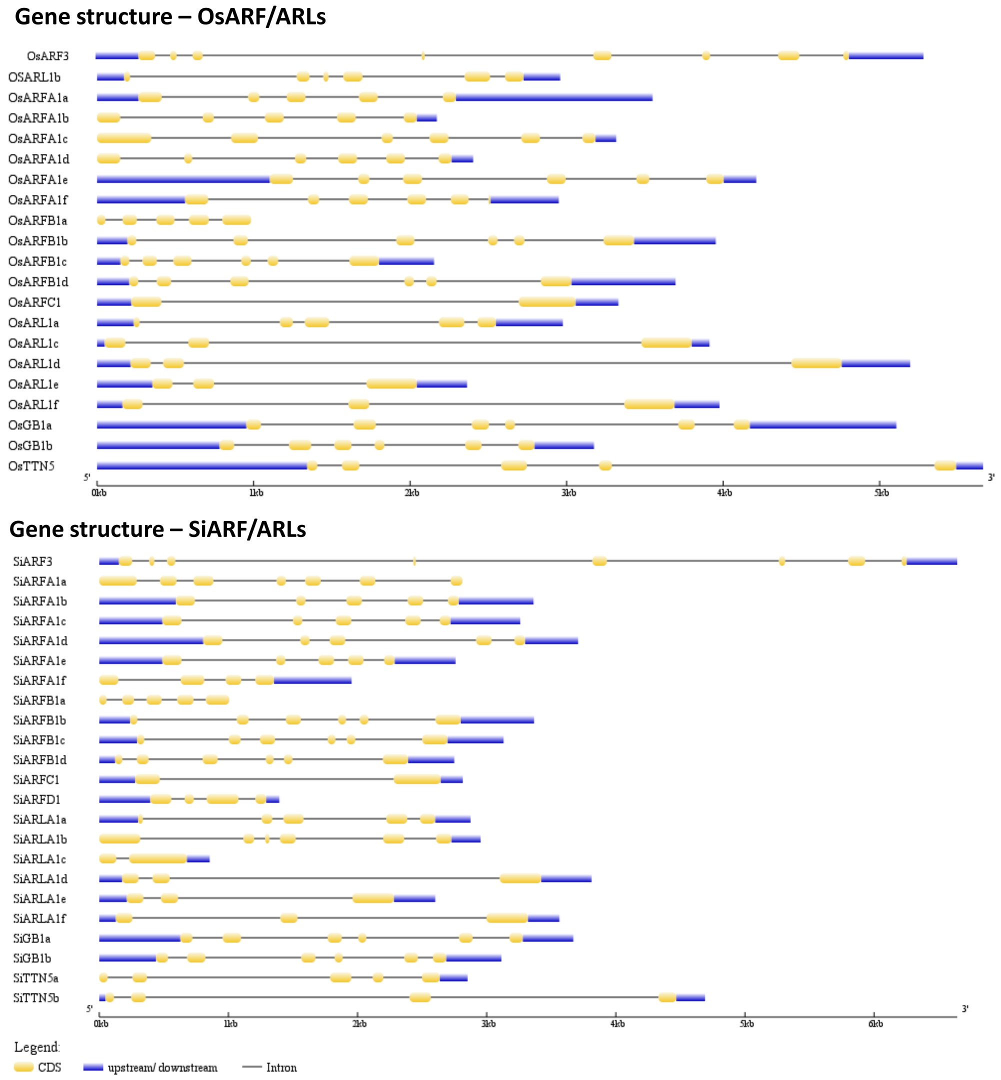

Supplementary Fig. S2. Gene ontology annotation data of OsARF and OsARL proteins showing the identified (a) biological process, (b) molecular function, and (c) cellular component.

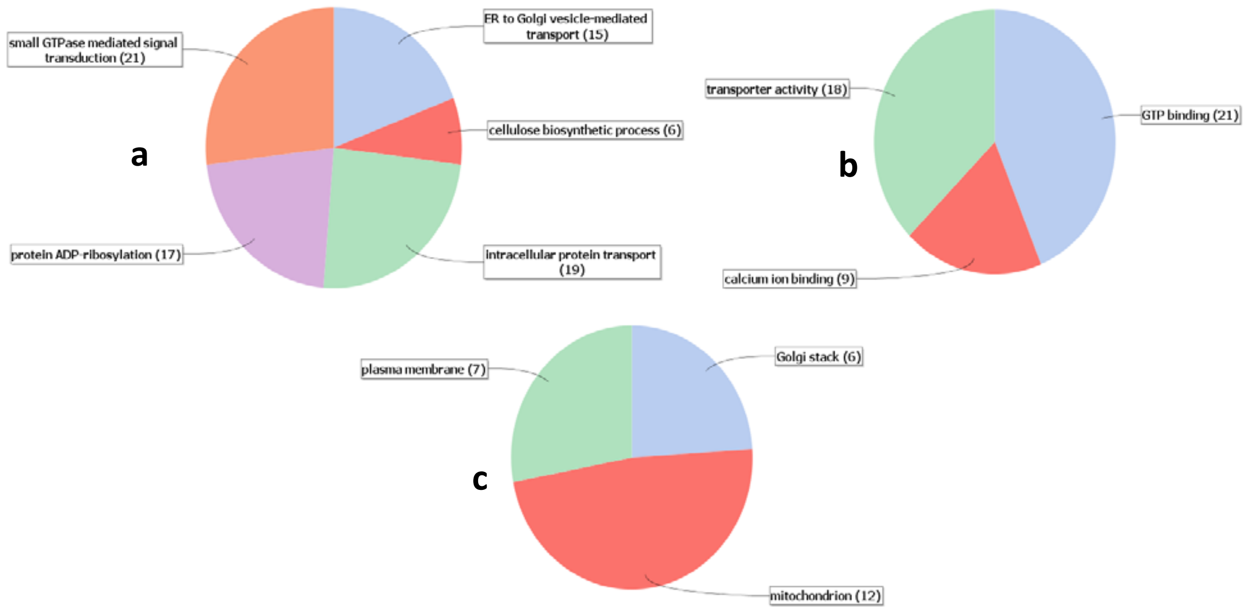

Supplementary Fig. S3. Relative expression of different gene members of the ARF family under various abiotic stresses as compared to the unstressed control.

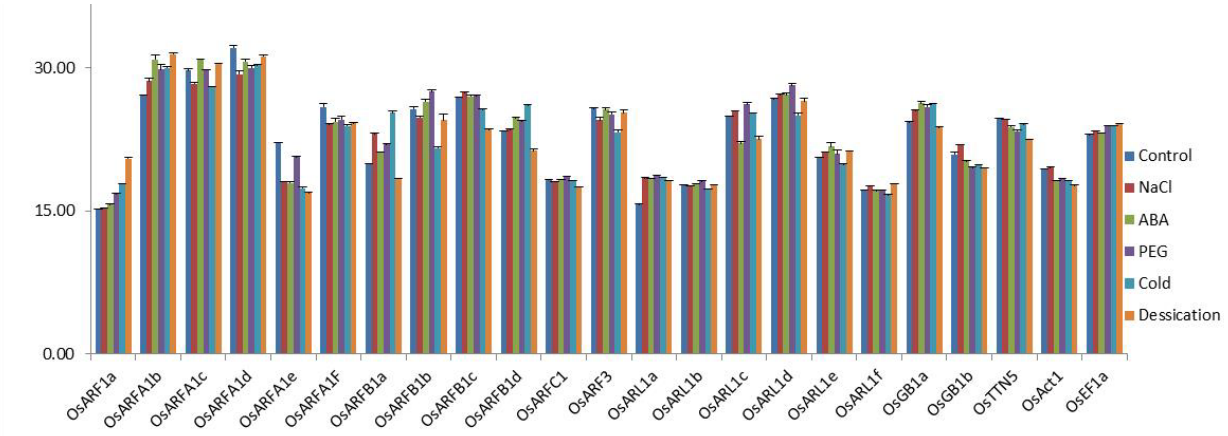

Supplementary Fig. S4. Alignment of *OsARFA1a, OsARFA1b, OsARFA1c, OsARFA1e, OsARFB1b, OsARFB1c* and *OsARFB1d* sequences of five rice cultivars, ‘Nipponbare’, ‘Vandana’, ‘N22’, ‘Azucena’ and ‘IR64’. Allelic variations were highlighted in blue color boxes. The light and dark shaded backgrounds indicate partial and entirely conserved amino acid residues, respectively.

OsARFA1a

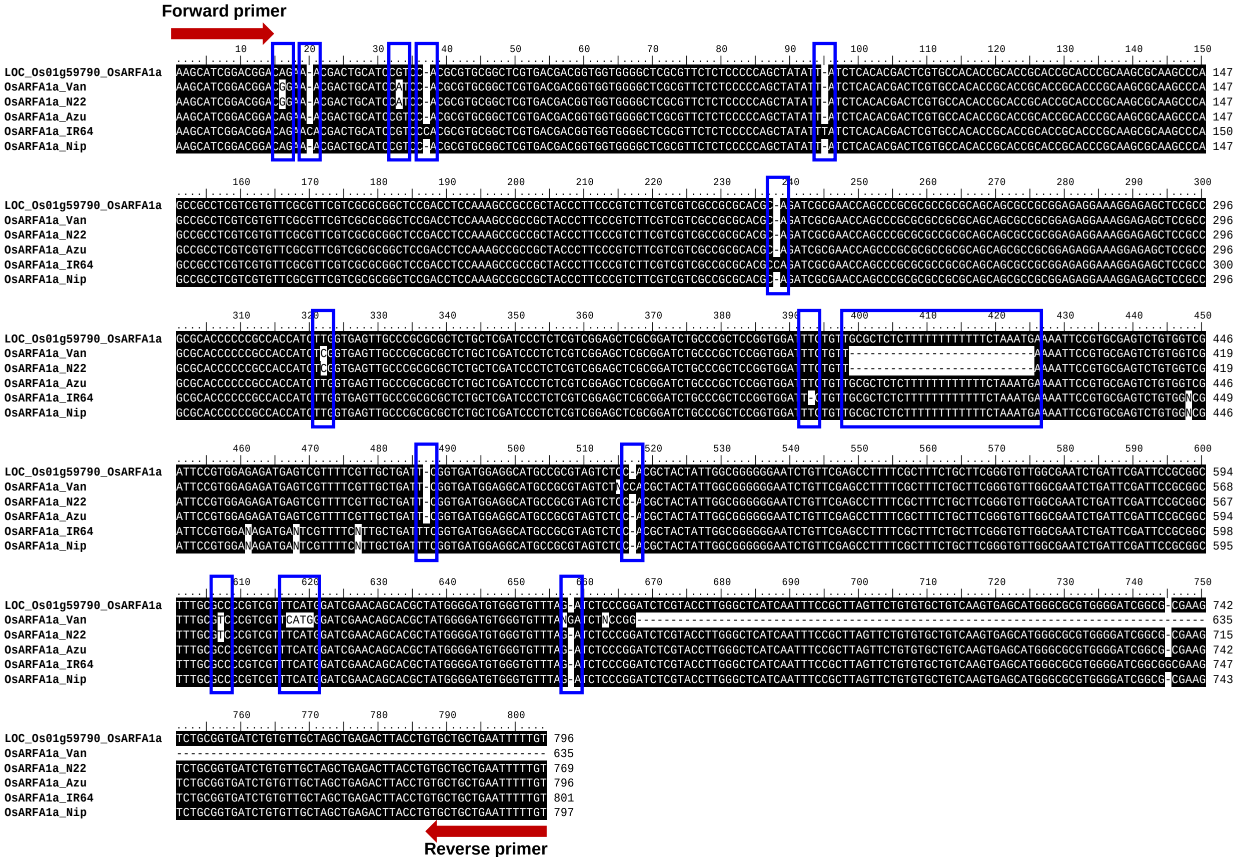

OsARFA1b

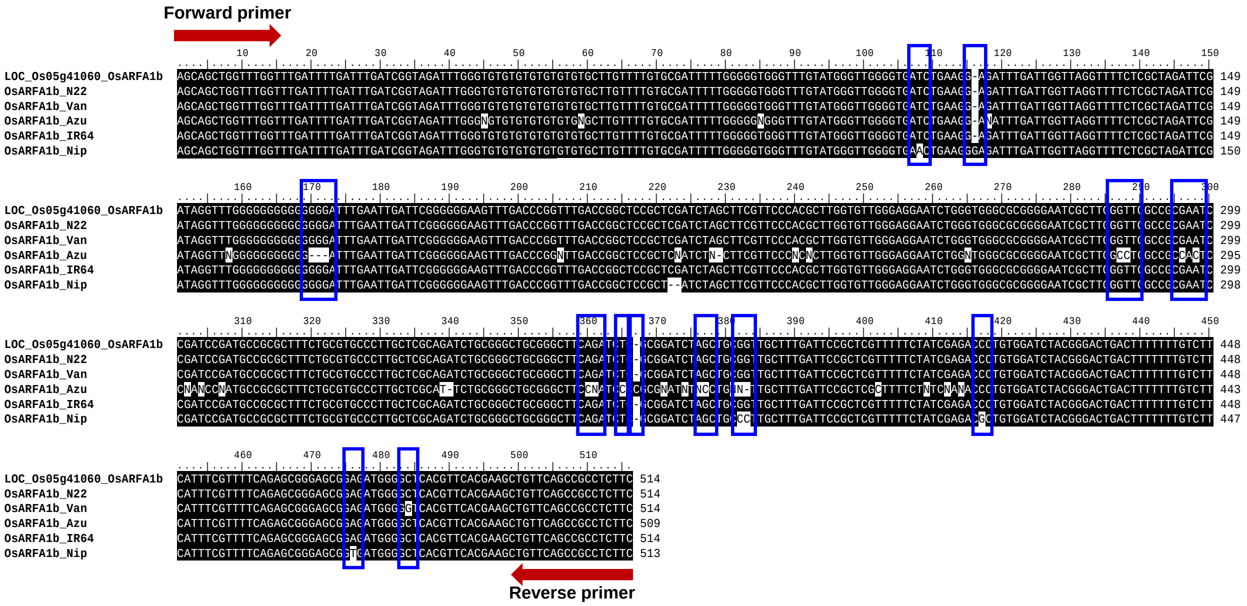

OsARFA1c

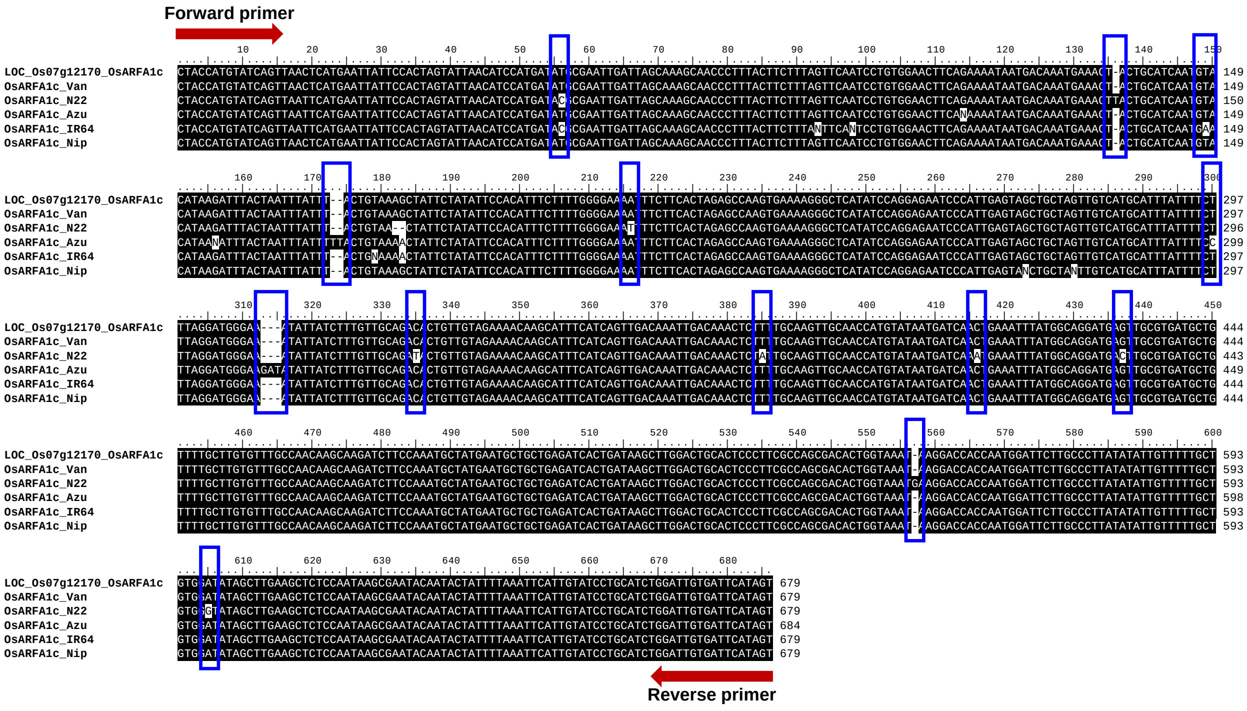

OsARFA1e

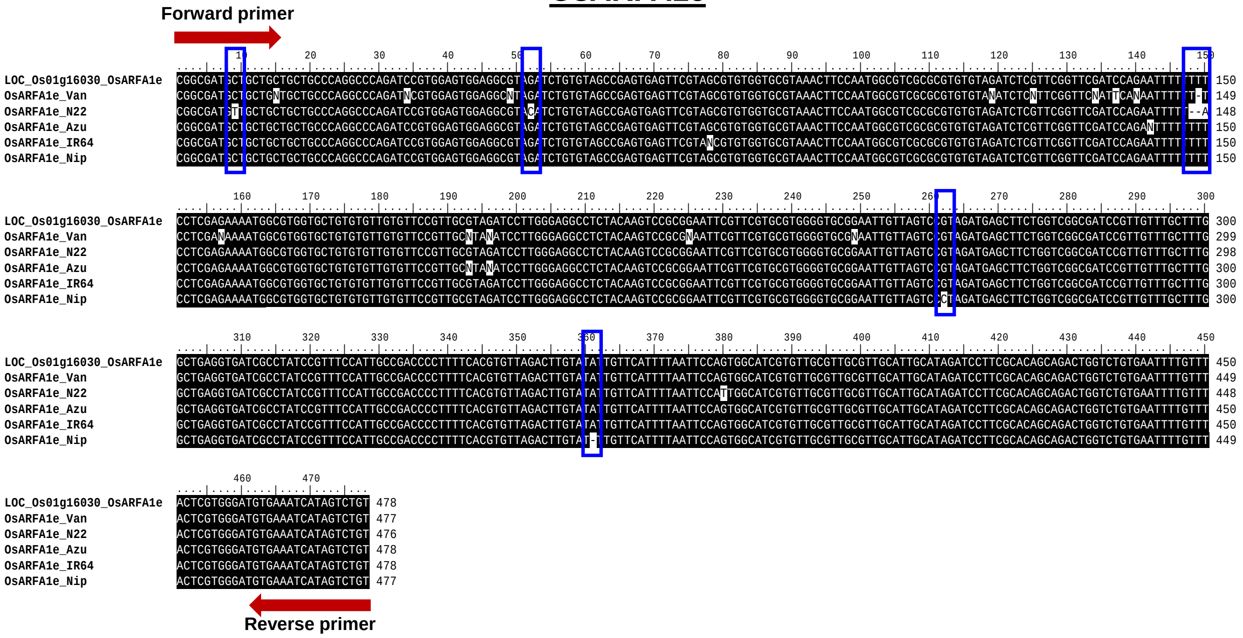
Forward primer
LOC_Os01g16030_OsARFA1e
OsARFA1e_Van
OsARFA1e_N22
OsARFA1e_Azu
OsARFA1e_IR64
OsARFA1e_Nip
Reverse primer

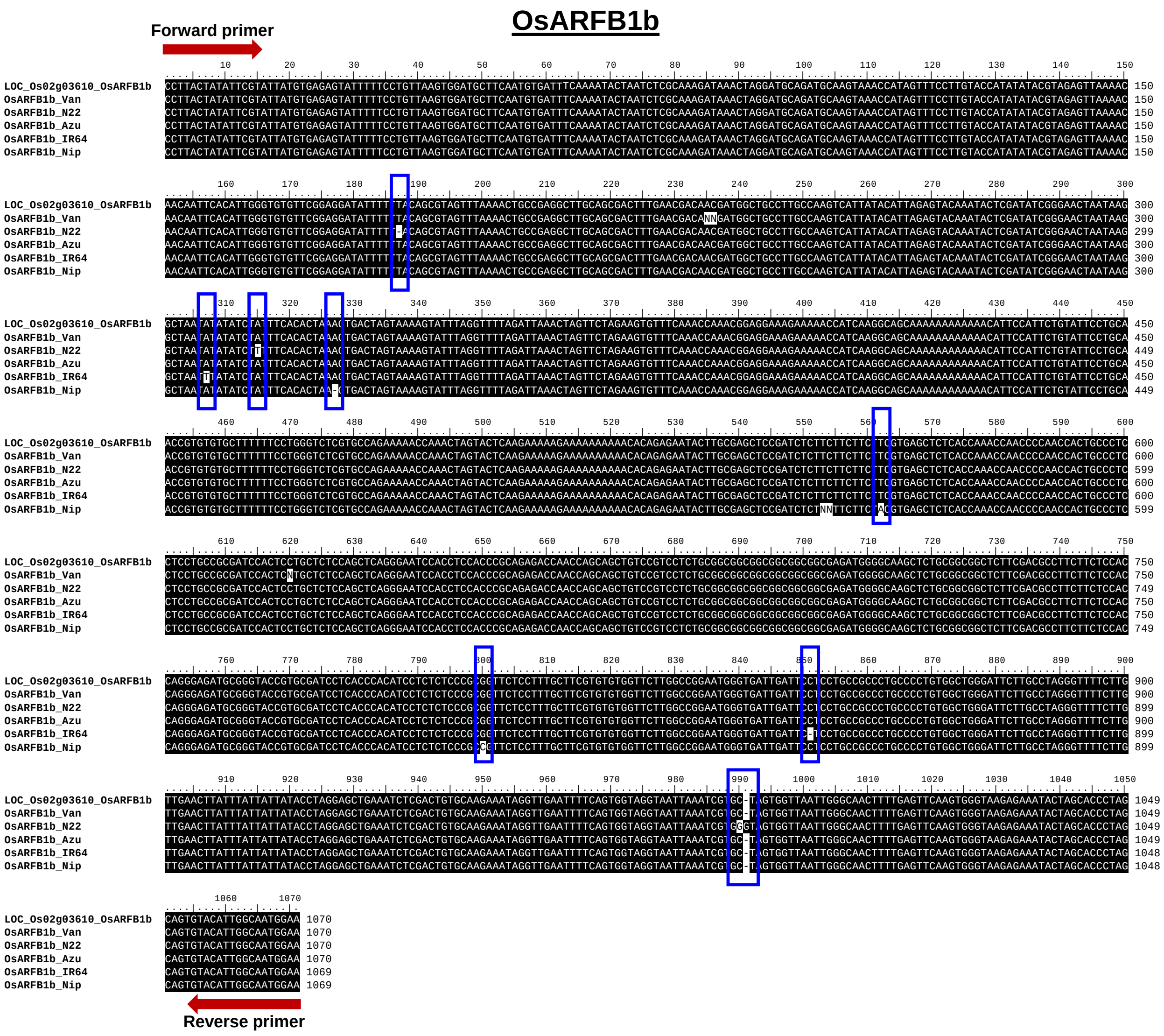
OsARFB1b
Forward primer
LOC_Os02g03610_OsARFB1b
OsARFB1b_Van
OsARFB1b_N22
OsARFB1b_Azu
OsARFB1b_IR64
OsARFB1b_Nip
Reverse primer

OsARFB1c

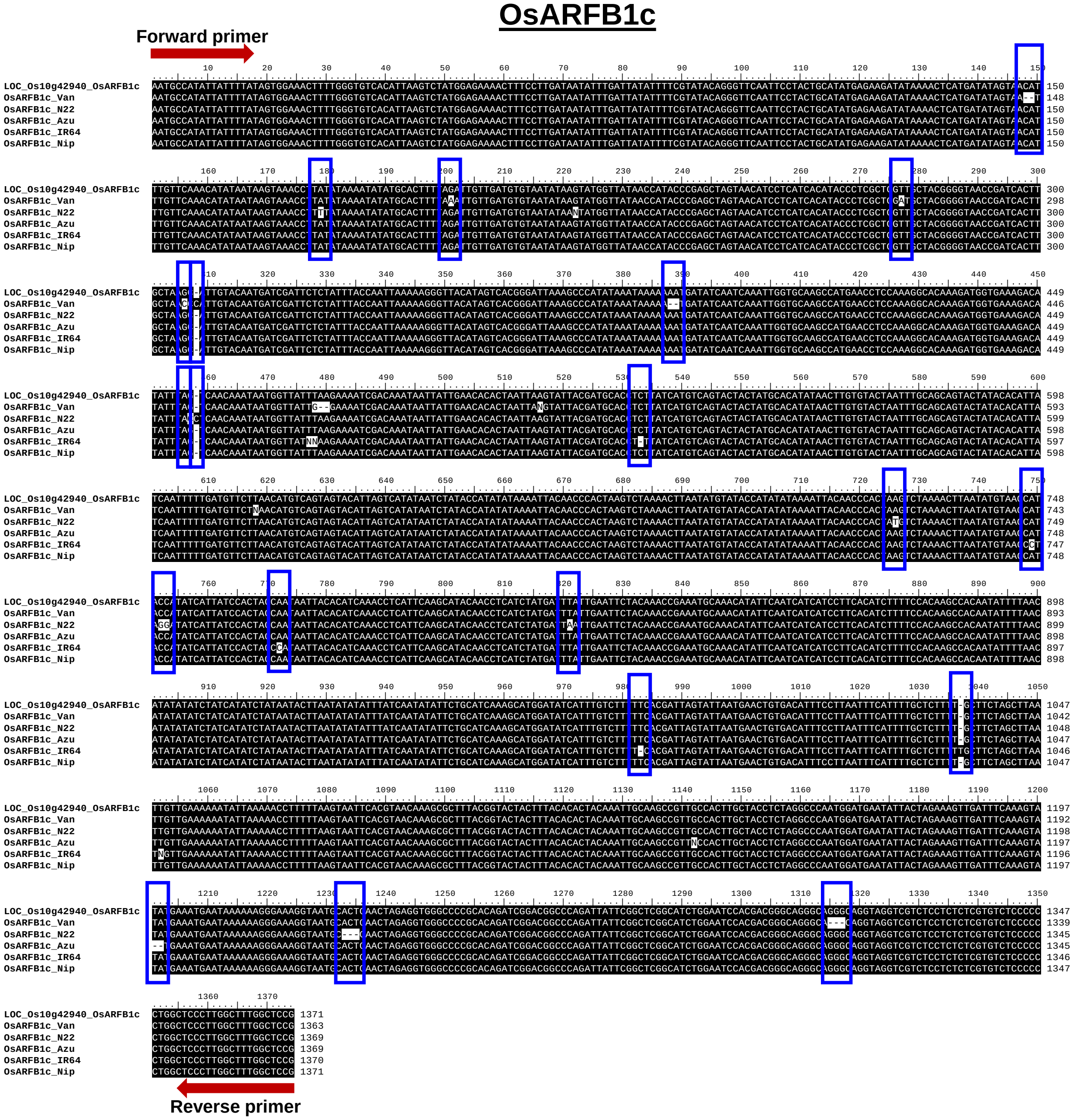

OsARFB1d

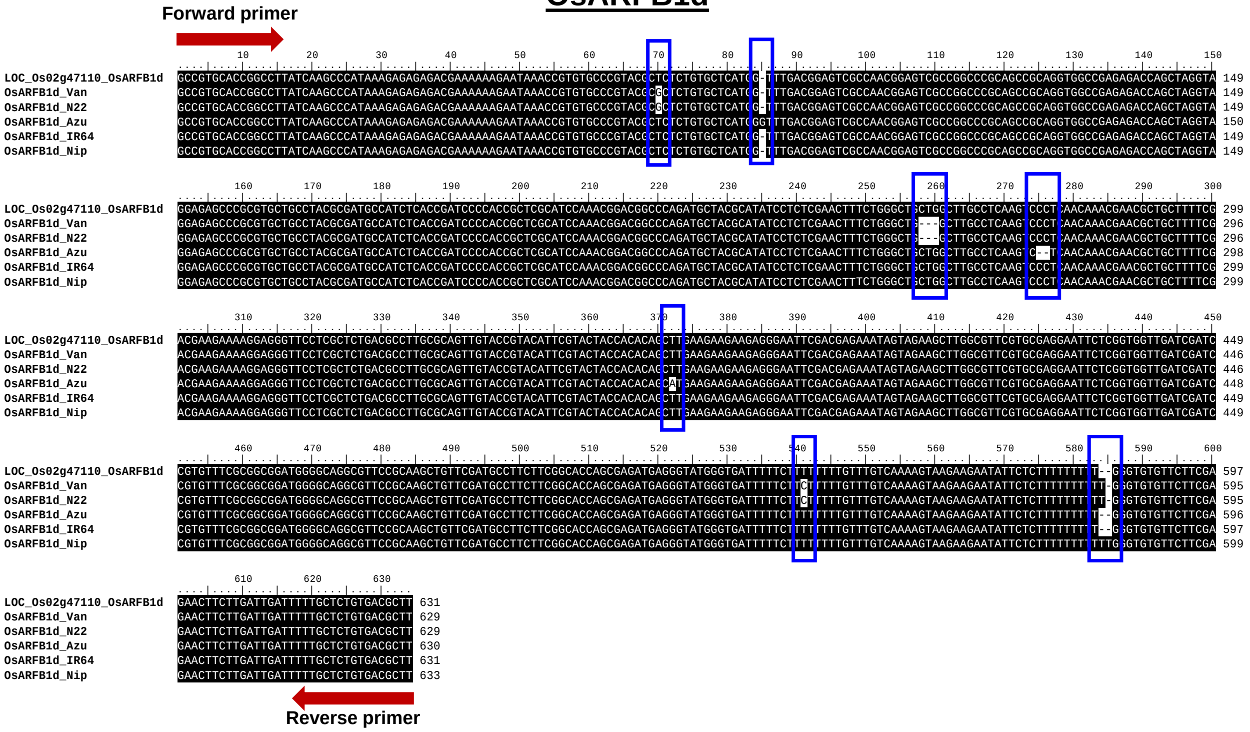
Forward primer
LOC_Os02g47110_OsARFB1d
OsARFB1d_Van
OsARFB1d_N22
OsARFB1d_Azu
OsARFB1d_IR64
OsARFB1d_Nip
Reverse primer

Supplementary Fig. S5. Pictorial representation of the salient findings derived from the analysis of ARF/ARL genes of rice and foxtail millet.

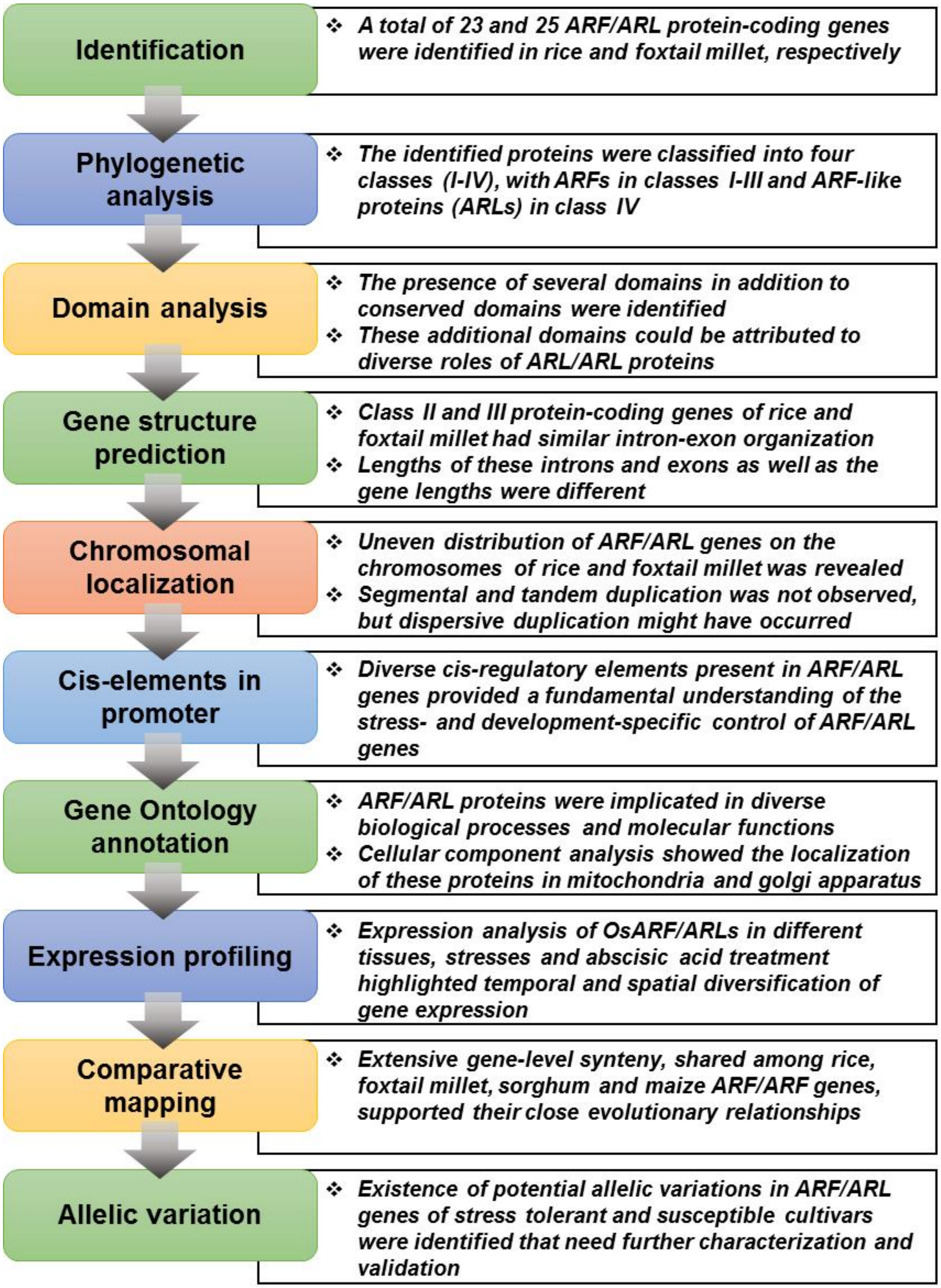

Supplement: Supplementary Information [file srep24008-s1.pdf]
